# Bioinspired nonheme iron complex that triggers mitochondrial apoptotic signalling pathway specifically for colorectal cancer cells†

**DOI:** 10.1039/d1sc05094j

**Published:** 2021-12-11

**Authors:** Yool Lee, Chaeun Oh, Jin Kim, Myong-Suk Park, Woo Kyun Bae, Kyung Hyun Yoo, Seungwoo Hong

**Affiliations:** Department of Chemistry, Sookmyung Women's University Seoul 04310 Korea hsw@sookmyung.ac.kr; Department of Biological Sciences, Sookmyung Women's University Seoul 04310 Korea khryu@sookmyung.ac.kr; Department of Chemistry, Sunchon National University Suncheon 57922 Korea; Division of Hemato-Oncology, Department of Internal Medicine, Chonnam National University Medical School and Hwasun Hospital Hwasun Republic of Korea drwookyun@jnu.ac.kr; Combinatorial Tumor Immunotherapy MRC Center, Chonnam National University Medical School Hwasun Republic of Korea

## Abstract

The activation of dioxygen is the keystone of all forms of aerobic life. Many biological functions rely on the redox versatility of metal ions to perform reductive activation-mediated processes entailing dioxygen and its partially reduced species including superoxide, hydrogen peroxide, and hydroxyl radicals, also known as reactive oxygen species (ROS). In biomimetic chemistry, a number of synthetic approaches have sought to design, synthesize and characterize reactive intermediates such as the metal-superoxo, -peroxo, and -oxo species, which are commonly found as key intermediates in the enzymatic catalytic cycle. However, the use of these designed complexes and their corresponding intermediates as potential candidates for cancer therapeutics has scarcely been endeavored. In this context, a series of biomimetic first-row transition metal complexes bearing a picolylamine-based water-soluble ligand, [M(HN_3_O_2_)]^2+^ (M = Mn^2+^, Fe^2+^, Co^2+^, Cu^2+^; HN_3_O_2_ = 2-(2-(bis(pyridin-2-ylmethyl)amino)ethoxy)ethanol) were synthesized and characterized by various spectroscopic methods including X-ray crystallography and their dioxygen and ROS activation reactivity were evaluated *in situ* and *in vitro*. It turned out that among these metal complexes, the iron complex, [Fe(HN_3_O_2_)(H_2_O)]^2+^, was capable of activating dioxygen and hydrogen peroxide and produced the ROS species (*e.g.*, hydroxyl radical). Upon the incubation of these complexes with different cancer cells, such as cervical, breast, and colorectal cancer cells (MDA-MB-231, AU565, SK-BR-3, HeLa S3, HT-29, and HCT116 cells), only the iron complex triggered cellular apoptosis specifically for colorectal cancer cells; the other metal complexes show negligible anti-proliferative activity. More importantly, the biomimetic complexes were harmless to normal cells and produced less ROS therein. The use of immunocytochemistry combined with western blot analysis strongly supported that apoptosis occurred *via* the intrinsic mitochondrial pathway; in the intracellular network, [Fe(HN_3_O_2_)(H_2_O)]^2+^ resulted in (i) the activation and/or production of ROS species, (ii) the induction of intracellular impaired redox balance, and (iii) the promotion of the mitochondrial apoptotic signaling pathway in colorectal cancer cells. The results have implications for developing novel biomimetic complexes in cancer treatments and for designing potent candidates with cancer-specific antitumor activity.

## Abstract 2

A water-soluble iron complex that produces hydroxyl radical species triggers colorectal cancer cell death *via* the mitochondrial apoptotic pathway.

## Graphical abstract

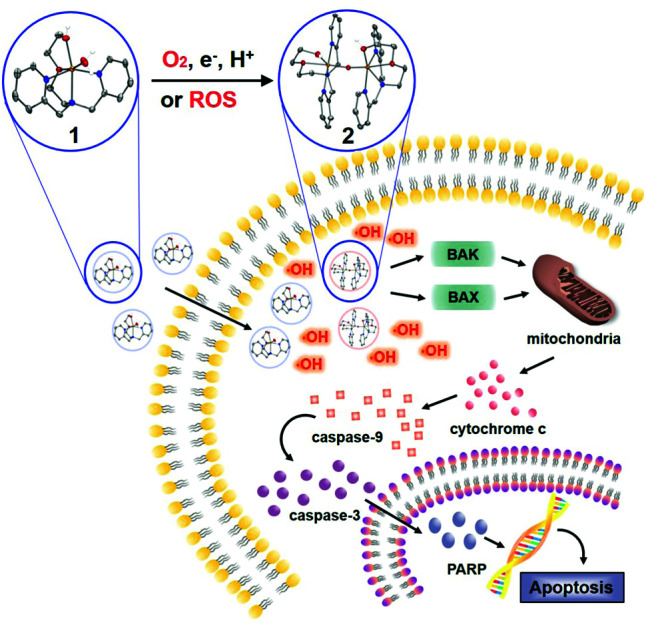

## Introduction

Cancer is one of the most harmful and serious heterogeneous diseases that represent abnormal cellular energy metabolism and remains one of the major causes of death in most developing and developed countries.^1^ Although extensive labours have been devoted to discovering targeted therapies, it is still challenging to overcome poor prognoses and high mortality. Thus, the pursuit of new chemical tools dealing with biomedical functions of metal complexes has garnered tremendous interest due to their broad pharmaceutical properties as potent anticancer agents. For instance, cisplatin, one of the most clinically successful examples of platinum-based anticancer drugs, opened a new era for the development of anticancer transition metal compounds by covering about 50% of all cancer treatments to date.^2–4^ Despite the outstanding applicability of Pt-based drugs, they suffer from low stability under physiological conditions resulting in copious toxic side effects such as necrosis, tissue injury, nausea, vomiting, and neurotoxicity.^5–8^ Ever since the pioneering work of the Au(i)–NHC complex was reported by Bernes-Price and co-workers in 2004,^9^ recent developments in the design of non-Pt-based metal N-heterocyclic carbene (NHC) complexes, such as those of Ru, Au, Ir, and Pd, have succeeded in the significant improvement of their stability due to the strong donating ability of the NHC ligand.^10–15^ Still, these metals are non-existing elements for the human body and may be the source of unexpected side effects along with the development of drug resistance.^16–19^ Therefore, the use of first-row transition metals such as Mn, Fe, Co, and Cu would be a credible alternative route for next generation anticancer agents with low general toxicity because they are bio-relevant trace elements. In particular, iron, a redox-active essential element involved in oxygen transport in mammals and electron transport in iron–sulfur proteins,^20–24^ would be expected to bypass the cytotoxicity concerns and function both as an electron donor and acceptor in order to disturb the redox homeostasis of the reactive oxygen species (ROS) level in tumors.^25^ However, the anticancer activity of first-row transition metal-based drugs have mostly been examined by introducing or vectorizing metal chelators as potential chemotherapeutics;^26–31^ their *in situ* mechanism of action and reactivity were scarcely scrutinized due to the intrinsic instability of reactive metal–oxygen intermediates.

Recent investigations have highlighted the importance of redox balance and the deregulation of redox signalling in cancer cells due to the raised levels of ROS from multiple intracellular factors such as increased metabolic activity, mitochondrial dysfunction, and peroxisome activity.^32–35^ On the contrary, ROS, which are by-products of normal cellular activity mainly generated in mitochondria and membrane-bound NADHP oxidase,^36,37^ are persistently produced in a highly controlled manner in normal cells because the canonical production of ROS is required for the signalling processes of cell division, autophagy, and cellular proliferation.^38,39^ Hence, a wealth of recent evidences have underlined ROS as a double-edged sword in cancer cells (*e.g.*, a cancer-stimulating or a cancer-suppressing agent). Such dichotomic role in cancer is also relevant to nitric oxide.^40,41^ Nevertheless, aiming ROS regulation for the clinical treatment of cancer represents a valuable challenge to advance cancer therapeutic approaches. In this context, we reasoned that the use of biomimetic metal complexes would (i) advance our understanding of the molecular basis of their mechanism of action, (ii) offer an elegant way to control intracellular ROS dysregulation, and (iii) provide a wealth of repertoire of metallodrugs having a broader spectrum of cancer-specific antitumor activity with a prudent choice of ligands.

**Scheme 1:**
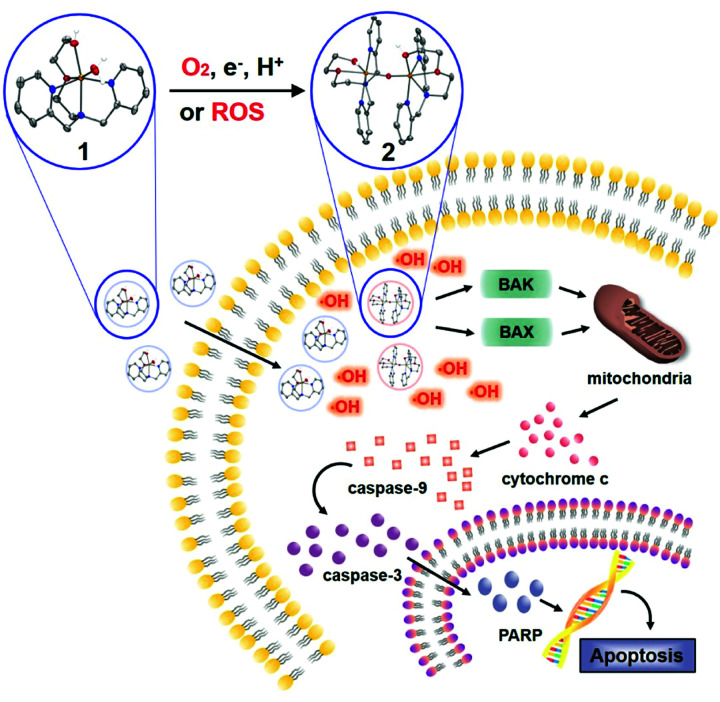
Proposed mitochondrial apoptotic pathway of colorectal cancer cells promoted by the iron complex.

In the course of our investigation, we employed biomimetic metal complexes having picolylamine-based water-soluble ligand, which accommodates first-row transition metals such as manganese, iron, cobalt, and copper. Their reactivity toward dioxygen and ROS was examined and then their anticancer activity was directly evaluated upon their incubation with cancer cells. It was found that the iron complex that was capable of activating both dioxygen and ROS triggered an efficient mitochondrial apoptotic signalling pathway of colorectal cancer cells by producing hydroxyl radicals and provoking the intrinsic mitochondrial apoptotic pathway (Scheme 1).

## Results and discussion

### Synthesis and characterization of first-row transition metal complexes

**Fig. 1:**
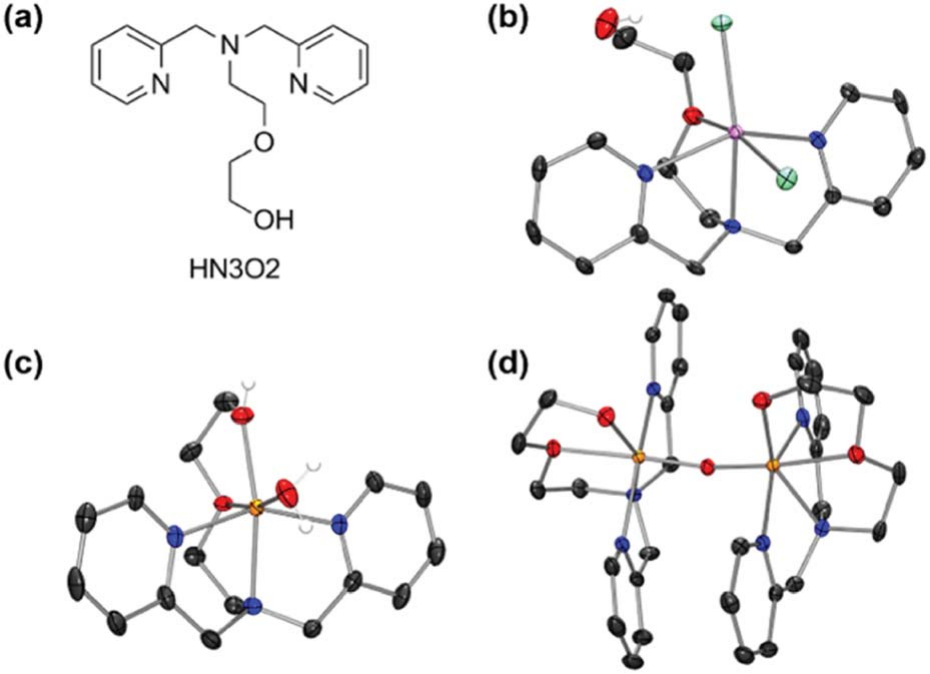
(a) Structure of ligand, HN_3_O_2_ and X-ray crystal structures of (b) [Mn(HN_3_O_2_)(Cl)_2_], (c) [Fe(HN_3_O_2_)(H_2_O)]^2+^, and (d) [Fe_2_(μ-O)(HN_3_O_2_)_2_]^4+^ with thermal ellipsoids showing 50% probability. Perchlorate ions and solvent molecules were omitted for clarity. Mn, pink; Fe, orange; Cl, aquamarine; N, blue; O, red; C, gray; H, white.

First-row transition metal complexes bearing a *N*,*N*-bis(2-picolyl)amine backbone with a pendant ethoxyethanol side chain (HN_3_O_2_), [M(HN_3_O_2_)]^2+^ (M = Mn, Fe, Co, and Cu) were synthesized according to the modified literature procedure (see ESI†).^42^ The X-ray crystal structures of manganese and iron complexes, [Mn(HN_3_O_2_)(Cl)_2_] and [Fe(HN_3_O_2_)(H_2_O)]^2+^ (1), were obtained when recrystallization was carried out under nitrogen atmosphere (Fig. 1a, b and Table S1†). As depicted in Fig. 1, both the iron(ii) and manganese(ii) centers in 1 and [Mn(HN_3_O_2_)(Cl)_2_], were coordinated to the HN_3_O_2_ ligand, which afforded a facial array of two pyridine N donors and one O donor from an ethoxy group; a water molecule or counterion completed the distorted octahedral coordination sphere. Electrospray ionization mass (ESI MS) spectra of 1 and [Mn(HN_3_O_2_)(Cl)_2_] displayed prominent peaks at *m*/*z* of 442.0 and 377.1, whose mass and isotopic distribution patterns correspond to [Fe(HN_3_O_2_)(ClO_4_)]^+^ and [Mn(HN_3_O_2_)(Cl)]^+^, respectively (Fig. S1†). Since a 2-His-1-carboxylate facial triad is a common feature of the active sites in dioxygen-activating nonheme metalloenzymes,^43–46^ we first explored the dioxygen activation reactivity of [M(HN_3_O_2_)]^2+^ under aerobic reaction conditions. Upon exposure to air, CH_3_CN-solution and an aqueous solution of [M(HN_3_O_2_)]^2+^ complexes did not show any spectral changes for several hours (Fig. S2 and S3†). This indicated that these metal complexes were stable in both organic and aqueous solution; they did not undergo demetallation, aggregation, and precipitation. Interestingly, only when 1 was recrystallized in a solvent mixture of CH_3_CN : ether (v/v 1 : 2) under aerobic conditions for several days, deep yellow single crystals due the formation of the μ-oxo-bridged diferric complex, [Fe_2_(μ-O)(HN_3_O_2_)_2_]^4+^ (2), was obtained (Fig. 1c and Table S2†). This suggested that among [M(HN_3_O_2_)]^2+^, only 1 was susceptible to conduct the dioxygen activation reaction depending on the reaction conditions (*vide infra*).^47,48^ From the charge consideration, the iron ions in 2 were both in the +3 oxidation state. Each Fe ion was at the center of a distorted N_3_O_3_ octahedron with one bridging oxo ligand forming a {Fe_2_(μ-O)} core with a Fe–O–Fe angle of 165.08(6)° (Table S2†). The Fe⋯Fe distance (3.530(1) Å) and the Fe–O distance (1.7799(4) Å) were consistent with a μ-oxo bridged diiron complex (Table S2†).^49–53^

### Reactivity of 1 toward dioxygen and hydrogen peroxide

**Fig. 2:**
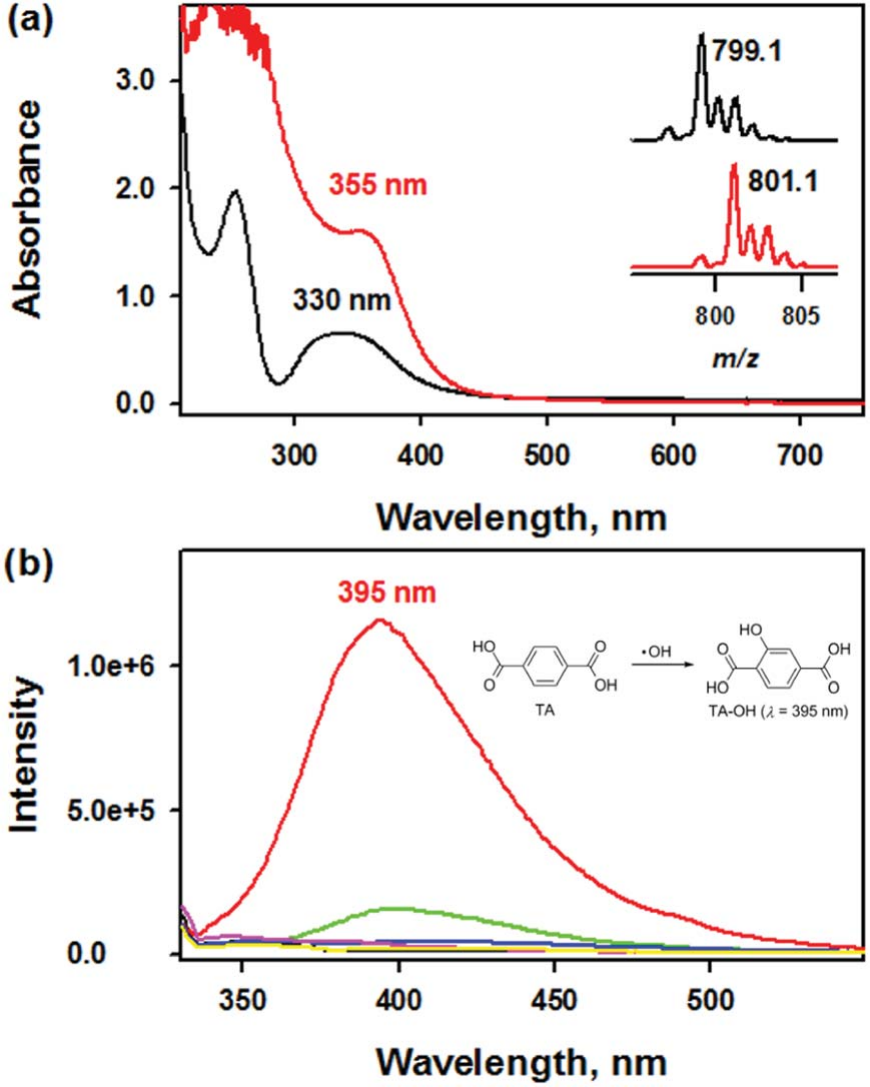
(a) UV-vis spectra of 1 (black line, 0.25 mM) and 2 (red line, 0.25 mM) in CH_3_CN at 20 °C. Insets show the isotopic distribution patterns of 2-^16^O and 2-^18^O (also see Fig. S5†). (b) Overlaid fluorescence spectra (*λ*_ex_ at 310 nm) obtained in the reaction of terephthalic acid (10 mM, black line) and ligand (green line) or [M(HN_3_O_2_)]^2+^ (M: Mn (blue line), Fe (red line), Co (pink line), Cu (yellow line)) in deionized water (DW) at room temperature after 24 h incubation. The HN_3_O_2_ ligand exhibits weak fluorescence at 395 nm in deionized water in the presence of TA at room temperature.

To provide more insight into the reactivity of complex 1, we scrutinized the reactivity of complex 1 toward dioxygen and hydrogen peroxide (H_2_O_2_). The UV-vis spectrum of isolated 2 clearly exhibited an intense absorption band at 355 nm (*ε* = 6000 M^−1^ cm^−1^) in CH_3_CN at 20 °C, which differed from that of 1 (Fig. 2a). Interestingly, when 1 was treated with iodosylbenzene (PhIO) or H_2_O_2_, 2 was generated instantaneously (Fig. S4†). The ESI MS spectrum of 2 clearly exhibited two prominent peaks at *m*/*z* of 350.1 and 799.1, whose mass and isotopic distribution patterns are in a good agreement with [Fe_2_(O)(N_3_O_2_)_2_]^2+^ and [Fe_2_(O)(N_3_O_2_)_2_(ClO_4_)]^+^, respectively (Fig. 2, inset and Fig. S5a†). The use of ^18^O-labeled PhIO resulted in two mass unit shift of the peak at *m*/*z* from 799.1 to 801.1, indicating that the oxygen atom is originated from PhI^18^O (Fig. 2, inset).

On the other hand, the addition of 1-benzyl-1,4-dihydronicotinamide (BNAH), known as an NADH analog, to an CH_3_CN-solution of 1, also afforded the formation of 2 in the presence of acid (*e.g.*, perchloric acid or hydrochloric acid) under aerobic conditions (Fig. S6†).^54^ The ESI MS spectrum of 2 generated upon the use of ^18^O_2_ evenly showed a peak at *m*/*z* 801.1, demonstrating that the source of the bridging oxygen atom was dioxygen (Fig. S5b†). Therefore, 1 is capable of activating dioxygen using biological ingredients (*e.g.*, proton and cofactors). The electrochemical property of 1 was examined by cyclic voltammetry. Despite an irreversible redox wave of 1, the reduction peak potential (*E*_red_) value of −0.15 V *vs.* Fc/Fc^+^ in CH_3_CN was found; when an identical experiment was performed in deionized water, the *E*_red_ value was positively shifted (*e.g.*, 0.08 V *vs.* Fc/Fc^+^) (Fig. S7†). According to the well-established results in the literature, the *E*_red_ value below ∼−0.1 V *vs.* Fc/Fc^+^ is defined to be a prerequisite value for dioxygen activation by nonheme iron(ii) complexes.^47,55^ Hence, 1 activates dioxygen with the help of a cofactor such as the NADH analog and proton under aerobic conditions or activates H_2_O_2_ (Fig. S4 and S6†); this results in the formation of the μ-oxo-bridged dinuclear iron complex.

Furthermore, we attempted to detect the generation of ROS species such as hydroxyl radical (·OH) due to 1 using fluorescence probe (*e.g.*, terephthalic acid (TA) assay).^56^ Upon incubation of 1 with non-fluorescent TA under aerobic conditions for 24 h, a brilliant fluorescence at 395 nm was detected by fluorescence spectrophotometry (*λ*_ex_ = 310 nm), indicating the hydroxylated product, 2-hydroxyterephthalic acid (TA-OH) was formed upon 24 h incubation (Fig. 2b). Indeed, over 50% generation of TA-OH in the reaction between TA and 1 can be achieved within 1 h under identical reaction conditions (Fig. S8†). Since TA, 1, and 2 did not show any fluorescence at 310 nm excitation wavelength, the appearance of the fluorescence intensity at 395 nm due to TA-OH solely comes from the reaction between 1 and TA under aerobic conditions. Interestingly, the incubation of other metal complexes or ligands only with TA did not afford such strong fluorescence (Fig. 2b). Therefore, the present result confirmed the efficacy of 1 for the *in situ* production of ·OH under aerobic conditions.

**Scheme 2:**
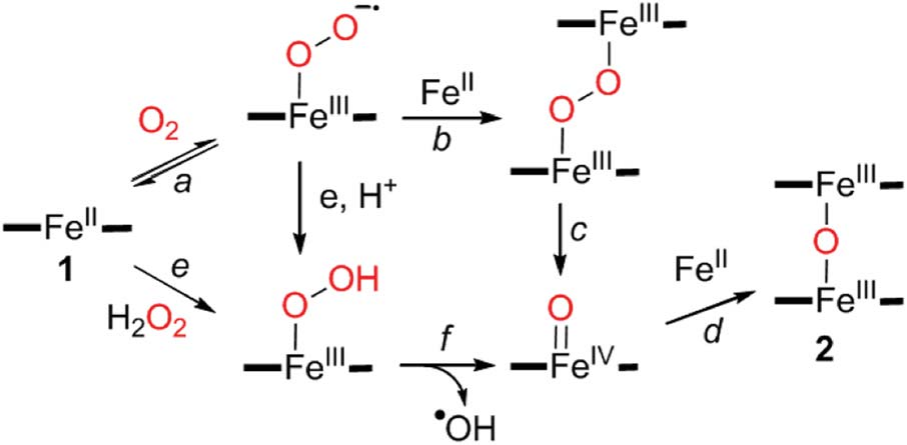
Proposed mechanism for the formation of the μ-oxo-bridged diferric complex *via* the reductive activation of dioxygen or hydrogen peroxide.

By virtue of well-established dioxygen activation mechanism,^47,55,57^1 could react with dioxygen to form a putative iron-superoxo species (Scheme 2, pathway a), which might further be converted to a μ-peroxo-bridged diferric species (Scheme 2, pathway b). Subsequent homolytic O–O bond cleavage results in the generation of iron-oxo species (Scheme 2, pathway c), followed by a comproportionation reaction with 1 to produce 2 (Scheme 2, pathway d). The addition of H_2_O_2_ might facilitate the formation of the iron-oxo species and produce the cytotoxic hydroxyl radical species as seen in the Fenton-like reaction (Scheme 2, pathway e and f).^58^

### Cell viability and intracellular ROS detection

**Fig. 3:**
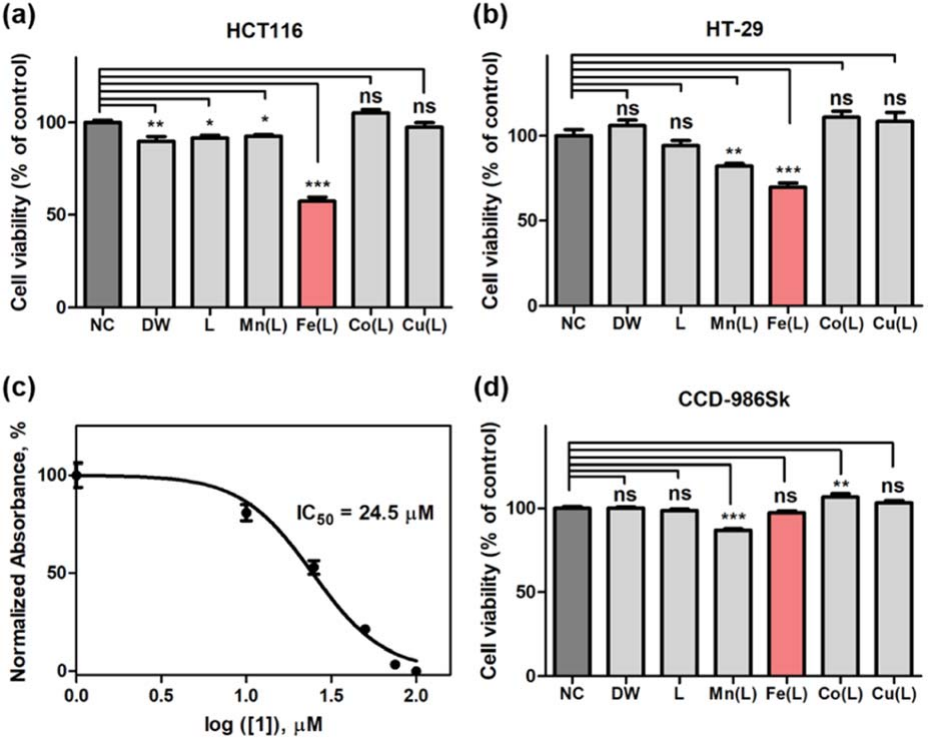
Effects of treatment of negative control (NC), deionized water (DW), ligand (L), and [M(HN_3_O_2_)]^2+^ (M(L); M = Mn, Fe, Co, and Cu) on (a) HCT116 and (b) HT-29. (c) Plot of normalized absorbance of HCT116 cells with respect to the logarithm concentration of 1 in order to determine the IC_50_ value. (d) CCD-986Sk cells on the total cell viability after 24 h by means of the WST-8 assay. The viability of cells without additional complexes is normalized to 100%. Statistical analysis was performed using one way ANOVA-Dunnett's test (* = *p* < 0.05, ** = *p* < 0.01, *** = *p* < 0.0001; ns = nonsignificant as compared to control).

Encouraged by the abovementioned dioxygen and ROS activation by the iron complex, we surmised that 1 could promote the deregulation of the intracellular ROS level and could induce cancer cell cycle arrest and cell death. Indeed, it has also been shown that the μ-oxo-bridged diiron(iii) complexes could exhibit tumor-specific anticancer activity by generating the toxic hydroxyl radical.^59–61^ The potential anti-proliferative activities of the above-mentioned biomimetic metal complexes against breast cancer cells (*e.g.*, MDA-MB-231, AU565, and SK-BR-3), cervical cancer cell (*e.g.*, HeLa S3), and colorectal cancer cells (*e.g.*, HT-29 and HCT116), were examined by the WST-8 assay (tetrazolium salt WST-8 = 2-(2-methoxy-4-nitrophenyl)-3-(4-nitrophenyl)-5-(2,4-disulfophenyl)-2*H*-tetrazolium, monosodium salt), which relies on the mitochondrial activity (Fig. 3 and S9†). Among biomimetic metal complexes, the iron complex, 1, only exhibits the anticancer activity toward the cancer cells, more specifically, the colorectal cancer cell lines such as HT-29 and HCT116; 1 is significantly effective to HCT116 than HT-29 (Fig. 3a and b). As shown in Fig. S10,†1 clearly revealed enhanced cytotoxicity against HCT116 cells while other metal complexes did not. The half-maximal inhibitory concentration (IC_50_) of 1 toward HCT116 cells is determined to be 24.5 μM (Fig. 3c). Such an IC_50_ value is greater than that of other reported Pt-based anticancer agents; however, it is still largely below the millimolar range, thereby showing significant cytotoxic activity against HCT116 cells.^62–64^ This result led us to, notably, the time-dependent WST-8 viability, which showed that the anticancer activity of 1 was observed with a clear viability decay up to ∼50% within 24 h while other metal complexes did not exhibit any kinetics of cytotoxicity on the HCT116 cells (Fig. S11†).

To show that 1 is not harmful to normal cells, we incubated 1 with CCD-986Sk cells, which are fibroblast cells for skin, under identical conditions. Very interestingly, all first-row transition metal complexes including 1 did not show any perceivable cell death (Fig. 3d). Contrary to other abiological metal-based anticancer drugs including Pt, Pd, and Ru that are cytotoxic by nature, these biomimetic metal complexes are significantly less toxic in the biological environment. These results strongly advocate that the use of the bio-relevant metal ions accommodating complexes might circumvent the side effects by better dealing with human physiological homeostasis.^65^

**Fig. 4:**
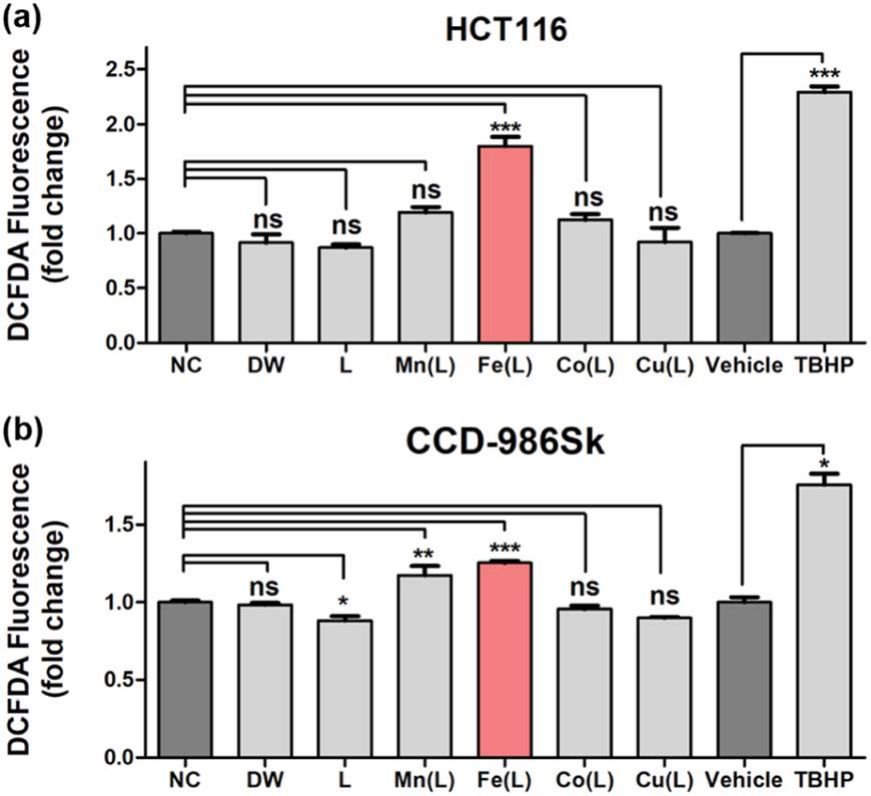
DCFDA assay for the detection of intracellular ROS produced in response to oxidative stress caused by DW, L, [M(HN_3_O_2_)]^2+^ (M(L); M = Mn, Fe, Co, and Cu), vehicle, and *tert*-butylhydroperoxide (TBHP) in (a) HCT116 and (b) CCD-986Sk cells. Cells were treated with 50 μM of metal complexes for 24 h and with 50 μM of TBHP alone for 4 h as the positive control. Data are shown as mean fold change (FC) to control ± standard error of the mean (SEM). The statistical analysis was performed using one way ANOVA-Dunnett's test and *t* test. Statistical significance is shown as: * = *p* < 0.05, ** = *p* < 0.01, *** = *p* < 0.001, ns = nonsignificant as compared to control (*n* = 3).

The qualitative and quantitative detection of intracellularly generated ROS by 1 in HCT116 cells and CCD-986Sk cells were carried out under identical incubation conditions using the cell permeant reagent 2′,7′-dichlorofluorescin diacetate (DCFDA) (Fig. 4). Consistent with previously demonstrated results with TA, only 1 effectively generated the ROS in HCT116 cells; the amount of intracellularly generated ROS was ∼1.8 fold greater than that observed in the negative control (NC) case (Fig. 4a). This result revealed that 1 efficiently penetrated into the HCT116 cells and produced intracellular ROS in the heterogeneous environment. Very importantly, the intracellular production of ROS by 1 in normal cells (*i.e.*, CCD-986Sk) was determined to be ∼1.2 fold greater (Fig. 4b). These observations allowed us to speculate that the intracellular generation of ROS by 1 may be favored in heterogeneous environment (*i.e.*, cancer cells) than in homogeneous environment (*i.e.*, normal cells). It has been demonstrated that an increase in ROS was associated with cancer cell growth as compared to normal cells and there might exist a certain threshold of ROS concentration that is incompatible with cellular survival.^66^ In the present case, we proposed that (i) 1 in HCT116 cells would activate intracellular ROS, (ii) provoke excessive levels of ROS that reaches a certain threshold, and (iii) finally exert cytotoxic effect. However, the less activation of ROS by 1 in normal cells would barely reach the threshold and the redox homeostasis would be maintained. Therefore, we concluded that once 1 was penetrated into the heterogeneous HCT116 cells, 1 increased the intracellular concentration of ROS in order to prompt the cell death by presumably affecting many regulatory signalling processes closely related to intracellular ROS homeostasis (*vide infra*).

### Immunocytochemistry, qRT-PCR, and western blot analysis

**Fig. 5:**
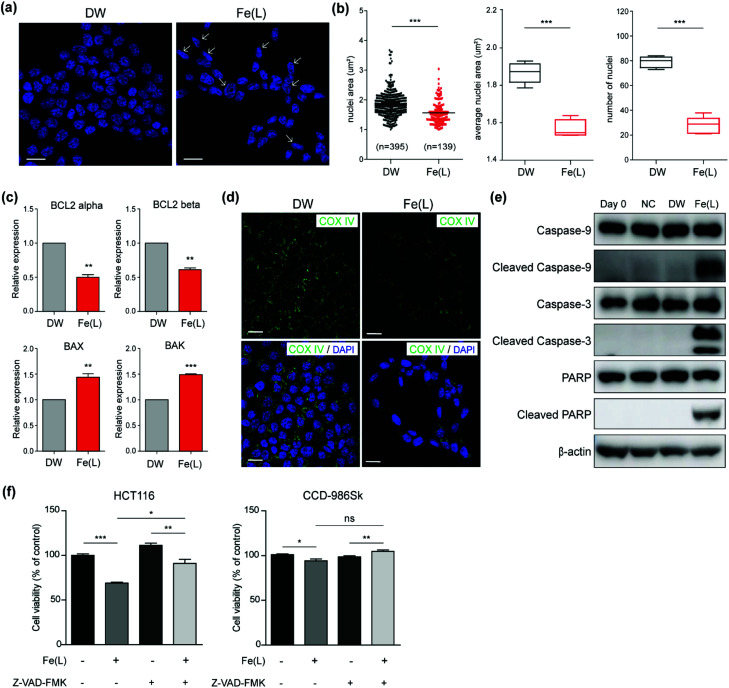
(a) Confocal images of nuclei stained with DAPI in HCT116 cells incubation with DW and 1 (50 μM) for 24 h. Scale bar: 20 μm. Arrows indicated nuclei with structural changes related to apoptosis. (b) Analysis of nuclei area and the number of nuclei measured by ImageJ. (c) Expression of BCL2 alpha, BCL2 beta, BAX, and BAK in HCT116 with DW and 1 (50 μM) for 24 h. (d) Confocal images of COX IV (green) and DAPI (blue) in HCT116 cells incubation with DW and 1 (50 μM) for 24 h. Scale bar: 20 μm. (e) Western blot analysis of caspase-9, 3 and PARP in HCT116 cells at day 0, negative control (NC), after incubation with deionized water (DW), and 1 (50 μM) for 24 h. (f) Cell viability of HCT116 and CCD-986Sk cells after 24 h was measured by the WST-8 assay. These cells incubated with or without 1 (50 μM) and Z-VAD-FMK (25 μM) for 24 h. The viability of cells without additional complexes is normalized to 100%. The statistical analysis was performed using two-tailed paired *t* test (* = *p* < 0.01, ** = *p* < 0.001, *** = *p* < 0.0001, ns = nonsignificant as compared to the control).

To verify that 1 roused the signalling pathway of cell death in colorectal cancer cells, we first performed immunofluorescence staining with 4′,6-diamidino-2-phenylindole (DAPI), which is frequently used to visualize the nuclear DNA in both living and fixed cells, in HCT116 cells within 24 h after treatment with 1 (Fig. 5a). The structural changes of the nuclei including nuclear condensation and shrinkage were noticed in HCT116 cells treated with 1; this result suggested the production of the apoptotic nuclei upon treatment with 1 (Fig. 5a, shown with a white arrow). We further examined the area and number of nuclei in HCT116 cells treated with 1 (Fig. 5b). A significant decrease in the nuclei area as well as the average nuclei area per image in HCT116 cells incubated with 1 as compared to the control were confirmed. In addition, a decreased number of nuclei also suggested that 1 has a critical role in anticancer activity.

In order to understand whether the anticancer effect of 1 influenced the apoptosis of HCT116 cells, we monitored the level of genes and proteins closely related to the apoptotic pathways. We first checked the expression of the BCL-2 family members using qRT-PCR (Fig. 5c). It is well-documented that the BCL-2 family is considered to be divided into anti-apoptotic and pro-apoptotic members according to their functions.^67^ Even though the expression of BCL-2 alpha and beta, known as anti-apoptotic members, was slightly attenuated, the expression of BAX and BAK, known as pro-apoptotic members, was increased and prominent in HCT116 cells treated with 1. This clearly suggested that 1 induced apoptotic cell death.

Since up-regulated BAX and BAK commonly concerned the release of cytochrome c into the cytosol by increasing the permeability of the mitochondrial membrane,^68^ we performed immunofluorescence for COX IV to ascertain whether 1-triggered apoptosis was mediated by the mitochondria. COX IV is an inner membrane mitochondrial marker and their level is increased at the early stage of apoptosis and then decreased after the release of cytochrome c into the cytosol during the apoptotic process.^69^ As shown in Fig. 5d, COX IV was remarkably reduced in HCT116 cells treated with 1, whereas its level was abundant in control. These results supported that the apoptosis of HCT116 cells caused by 1 might be accompanied by a decrease in the mitochondrial function. Since it has been well-established that the decrease in the level of COX IV affected the activation of caspases, for instance, caspase 9 and 3,^69^ we examined the level of the cleaved caspase 9 and 3 after incubating HCT116 cells with 1 for 24 h. Western blot analysis undoubtedly showed that the level of the cleaved caspase 9 and 3 were remarkably increased in HCT116 cells (Fig. 5e). Moreover, an increased level of the cleaved poly(ADP-ribose) polymerase 1 (PARP1), which is a hallmark of apoptosis due to the caspases,^70^ was noticeably perceived in HCT116 cells treated with 1 (Fig. 5e).

Next, to investigate whether 1-triggered apoptosis was mediated by the extrinsic pathway through FADD (fas-associated protein with death domain), immunofluorescence for E-cadherin, which collaborates with death receptors, was conducted (Fig. S12 and S13†).^71,72^ We observed that there was no significant change in the E-cadherin level after treatment with 1. In addition, the expression of BID, which is proteolytically activated by FADD signalling, was not significant between HCT116 cells treated with 1 and the controls (Fig. S14†). These results supported that the apoptosis induced by 1 was presumably mediated by the intrinsic pathway including mitochondrial dysfunction but not by the extrinsic pathway. In addition, we performed the cell viability assay in the presence and absence of Z-VAD-FMK to evaluate the apoptotic effect of 1 on HCT116 cells. Z-VAD-FMK is a cell permeable caspase inhibitor that impedes caspase processing and apoptosis by irreversibly binding to the catalytic site.^73^1 induced cell death only in HCT116 and co-treatment with Z-VAD-FMK partly improved the viability level (Fig. 5f). CCD-986Sk is not affected by 1 and Z-VAD-FMK. These results showed that 1 was closely associated with the apoptosis-dependent pathway.

Taken together, we propose that after cellular uptake, the intracellular generation of ROS by 1 prompted mitochondrial dysfunction and resulted in the apoptotic signalling pathway as follows: (i) the mitochondrial release of cytochrome c in cytosol, followed by (ii) the cleavage of caspase 9 and then that of caspase 3 occurred to finally (iii) stimulate the cleavage of PARP1. Thus, the intrinsic mitochondrial apoptosis pathway was activated to cause HCT116 cell cycle arrest upon treatment with 1.

### *In vivo* studies

**Fig. 6:**
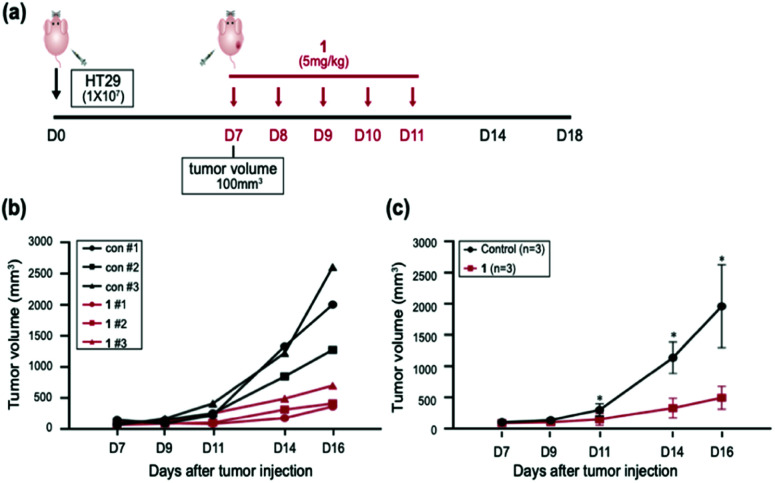
Inhibition of tumor growth by treatment of 1 in the subcutaneous colorectal cancer model. (a) Balb/c-nu mice were injected with 1 × 10^7^ HT29 cells subcutaneously in their right flank on day 0 of the experiment. Treatments with 1 or sterile distilled water control were injected intraperitoneally on days 7, 8, 9, 10 and 11. (b) Tumor growth curves were obtained for each individual mouse treated with either control (black triangle, rectangle and circle) or 1 (red triangle, rectangle and circle). (c) The results depict the mean ± standard error of mean (SEM) of the subcutaneous tumor volumes (*n* = 3 mice per group). *p < 0.05. Error bars represent SEM.

Having established that 1 elevated the intracellular ROS concentration, we investigated the anticancer activity of 1*in vivo*. To evaluate the anticancer effect of 1*in vivo*, six mice models injected with HT29 cells were used; three were used as the vehicle with sterile distilled water and another three were treated with 1 intraperitoneally for 5 days (Fig. 6a). When the mice were treated with 5 mg kg^−1^ of 1, no adverse side effects were detected. Three cases of 1 resulted in approximately 50% to 80% tumor growth inhibition with respect to the control (Fig. 6b). Furthermore, the injection of 1 at 5 mg kg^−1^ significantly inhibited the tumor growth of the xenograft relative to the control in the subcutaneous colorectal cancer model, indicating that 1 is a potential antitumor agent that operates *in vivo* (Fig. 6c). These results supported that 1 has the possibility of therapeutic target *in vivo*.

## Conclusions

In conclusion, we successfully synthesized and characterized a series of biomimetic first-row transition metal complexes with various spectroscopic methods including X-ray crystallography. Among these chemically designed complexes, only iron complex, 1, was capable of activating dioxygen and reactive oxygen species and producing hydroxyl radical species. This illustrated the importance of the choice of the redox active metal ion at the center of the biomimetic complex in order to judiciously attribute their chemical functionality and reactivity. This interesting chemical property of 1 was believed to trigger the cell cycle arrest of the cancer cells, more specifically colorectal cancer cells, by efficiently producing intracellular ROS in a heterogeneous environment. Strikingly, when normal cells were treated with 1 under identical conditions, cell death did not occur due to the less production of ROS under homogeneous environment. The accumulated evidences presented in the cell viability assays clearly supported the effective intracellular generation of ROS by 1 as compared to other biomimetic metal complexes. More detailed investigation on the signaling pathway of cell cycle arrest revealed that the intrinsic mitochondrial apoptotic pathway was activated by 1 through (i) the release of cytochrome c, (ii) the cleavage of caspase 9 and caspase 3, and (iii) the cleavage of PARP1 located in the nucleus. Finally, we confirmed the anticancer effect of 1 both *in vitro* and *in vivo* as the inhibition of tumor growth by 1 in the subcutaneous colorectal cancer model was verified. Overall, the present strategy (*e.g.*, the use of biomimetic metal complexes) might tremendously reduce the occurrence of off-target effects, thereby proving the possibility of a wide range of biological application and justify further investigation. Moreover, this would be an important asset—or, at least one of the central functions—that the designed biomimetic metal complexes merit more attention in the future direction of the cancer therapeutic research.

## Materials and methods

### Materials

Commercially available chemicals were used without further purification unless otherwise indicated. Solvents were dried according to the published procedures and distilled under Ar prior to use.^74^ H_2_^18^O (95% ^18^O-enriched) was purchased from ICON Services Inc. (Summit, NJ, USA). Iodosylbenzene (PhIO) was prepared by a literature method.^75^ The nonheme manganese, iron, cobalt, copper(ii) complexes were prepared according to the modified literature methods.^42^

### Instrumentation

The UV-vis spectra were recorded on a Hewlett Packard Agilent Cary 8454 UV-visible spectrophotometer equipped with a T2/sport temperature-controlled cuvette holder. Electrospray ionization mass spectra (ESI MS) were collected on a Thermo Finnigan (San Jose, CA, USA) LTQ™ XL ion trap instrument by infusing the samples directly into the source at 5.0 μL min^−1^ using a syringe pump. The spray voltage was set at 4.7 kV and the capillary temperature at 120 °C. Electrochemical measurements were performed on a CHI617B electrochemical analyzer (CH Instruments, Inc.) in CH_3_CN containing 0.10 M Bu_4_NPF_6_ (TBAPF_6_) or in H_2_O containing 0.10 M NaClO_4_ as the supporting electrolytes at 25 °C. A conventional three-electrode cell was used with a glassy carbon working electrode (surface area of 0.030 cm^2^), a platinum wire as the counter electrode, and an Ag/Ag^+^ electrode as the reference electrode (in CH_3_CN) or an Ag/AgCl, 1.0 M KCl electrode as a reference electrode (in H_2_O), respectively. The glassy carbon working electrode was routinely polished with BAS polishing alumina suspension and rinsed with acetone and acetonitrile before use. The measured potentials were recorded with respect to an Ag/Ag^+^ (0.010 M) reference electrode (in CH_3_CN) or an Ag/AgCl, 1.0 M KCl reference electrode (in H_2_O), respectively. All potentials (*vs.* Ag/Ag^+^ or Ag/AgCl) were converted to values *vs.* Fc/Fc^+^ by subtracting 0.09 V or 0.02 V, respectively.^76^

For the measurement of cell viability, CCK-8 solution from Enzo (Farmingdale, NY, USA) with the culture media was added to each well and the absorbance at 450 nm was measured by Synergy™ HTX Multi-Mode Microplate Reader from Bio-Tek (Winooski, VT, USA) and VICTOR Nivo™ Multimode Plate Reader on a PerkinElmer (Waltham, MA, USA). Chemiluminescent signals were induced by chemiluminescent detection reagent from ATTO (Taito, Tokyo, Japan) and detected by Amersham Imager 600 from GE Healthcare (Chicago, IL, USA). The images observed with a LSM 700 laser scanning confocal microscope and Axio Vert. A1-inverted microscope from Zeiss (Oberkochen, Baden-Württemberg, Germany) and analyzed using ImageJ from NIH (Bethesda, Maryland, USA). qRT-PCR was measured by LightCycler 96 Instrument from Roche (Basel, Basel-Stadt, Switzerland).

### Synthesis and characterization of mononuclear manganese(ii), iron(ii), cobalt(ii), and copper(ii) complexes

[Mn^II^(HN_3_O_2_)(Cl)_2_], [Fe^II^(HN_3_O_2_)(H_2_O)]^2+^ (1), [Co^II^(HN_3_O_2_)]^2+^ and [Cu^II^(HN_3_O_2_)]^2+^, were prepared by reacting HN_3_O_2_ ligand and equimolar MnCl_2_, Fe(ClO_4_)_2_·6H_2_O, Co(NO_3_)_2_·6H_2_O, and Cu(NO_3_)_2_·6H_2_O, respectively, in CH_3_CN.^42^ The UV-vis spectra of 1, [Mn^II^(HN_3_O_2_)(Cl)_2_], [Co^II^(HN_3_O_2_)]^2+^ and [Cu^II^(HN_3_O_2_)]^2+^, exhibited characteristic shoulder absorption bands at 330 nm for 1, about 300 nm for [Mn^II^(HN_3_O_2_)(Cl)_2_], [Co^II^(HN_3_O_2_)]^2+^, and 600 nm for [Cu^II^(HN_3_O_2_)]^2+^, respectively, in CH_3_CN at 20 °C.

### Synthesis and characterization of μ-oxo-Bridged diferric complex

The μ-oxo bridged diferric complexes bearing the HN_3_O_2_HN_3_O_2_ ligand, [Fe_2_(O)(HN_3_O_2_)_2_(ClO_4_)] (2), was prepared by three different ways: (i) by exposing 1 under aerobic conditions, (ii) by reacting 1 with excess solid iodosylbenzene (PhIO) or H_2_O_2_, (iii) by treating 1 with 1-benzyl-1,4-dihydronicotinamide (BNAH) in the presence of perchloric acid (1.0 equiv.) in CH_3_CN at 20 °C. The UV-vis spectrum of 2 exhibited a strong charge transfer band at 355 nm (*ε* = 6000 M^−1^ cm^−1^). The ESI MS spectrum of 2 displayed a prominent peak at *m*/*z* of 799.1, whose mass and isotopic distribution patterns are in a good agreement with [Fe_2_(O)(N_3_O_2_)_2_(ClO_4_)]^+^. ^18^O-labeled PhIO was prepared by incubating PhIO with H_2_^18^O (20 μL) for 20 min. The use of PhI^18^O resulted in the formation of [Fe_2_(^18^O)(N_3_O_2_)_2_(ClO_4_)]^+^, which appeared at *m*/*z* of 801.1 in the ESI MS spectrum.

### X-ray structural analysis

Single crystals of [Mn(HN_3_O_2_)(Cl)_2_], 1, and 2 suitable for X-ray crystallographic analysis were obtained by the slow diffusion of Et_2_O into a CH_3_CN solution of each metal complex. These crystals were taken from the solutions by a nylon loop (Hampton Research Co.) on a handmade cooper plate and mounted on a goniometer head in a N_2_ cryostream. The diffraction data for [Mn(HN_3_O_2_)(Cl)_2_], 1, and 2 were collected at 120 K on a Bruker SMART AXS diffractometer equipped with a monochromator in the Mo *K*α (*λ* = 0.71073 Å) incident beam. The cell parameters were determined and refined by the SMART program.^77^ The CCD data were integrated and scaled using the Bruker-SAINT software package.^78^ An empirical absorption correction was applied using the SADABS program.^79^ The structures were solved by direct methods, and all non-hydrogen atoms were subjected to anisotropic refinement by full-matrix least squares on *F*^2^ using SHELXTL Ver. 6.14.^80^ The crystallographic data and selected bond distances and angles are listed in Tables S1 and S2,† respectively.

### Dioxygen and hydrogen peroxide activation reactivity

Kinetic measurements were performed on a Hewlett Packard 8453 photodiode-array spectrophotometer for dioxygen and H_2_O_2_ activation reactions by 1 in CH_3_CN at 20 °C. Reactions were run in a 1.0 cm UV cuvette, monitoring the UV-vis spectral changes of the reaction solutions. The stability of the metal complexes were determined by monitoring the absorption band at 330 nm for 1, about 300 nm for [Mn^II^(HN_3_O_2_)(Cl)_2_], [Co^II^(HN_3_O_2_)]^2+^, and 600 nm for [Cu^II^(HN_3_O_2_)]^2+^, respectively, in CH_3_CN and H_2_O at 20 °C. The formation of 2 was detected by monitoring the absorbance at 355 nm in CH_3_CN at 20 °C. The kinetic experiments were run at least in triplicate, and the data reported represent the average of these reactions.

### Cell culture

The human carcinoma-derived cells such as HCT116 and HT-29, colorectal cancer cell line, MDA-MB-231 and AU565, breast cancer cell line and HeLa S3, cervical cancer cell line were maintained as the monolayer culture in the Roswell Park Memorial Institute (RPMI) 1640 medium, and SK-BR-3, breast cancer cell line was maintained in Dulbecco's Modified Eagle Medium (DMEM), and CCD-986Sk, Fibroblast cells were cultured in Iscove's Modified Dulbecco's Medium (IMDM) supplemented with 10% fetal bovine serum (FBS) and 1.0% penicillin-streptomycin. Cells were grown at 37 °C in a humidified atmosphere containing 5.0% CO_2_.

### Cell viability

To determine cell viability, cells were seeded in 96-well plates and allowed to recover for 24 h. Then, the cells were exposed to the metal complexes for 24 h. The metal complexes were dissolved in distilled water and diluted in fresh culture medium (final metal complex concentration is 50 μM). The added concentration of Z-VAD-FMK was 25 μM (Selleck Chem. #S7023). After 24 h treatment of the complexes, the cytotoxicity of the metal complexes was determined using the 2-(2-methoxy-4-nitrophenyl)-3-(4-nitrophenyl)-5-(2,4-disulfophenyl)-2*H*-tetrazolium monosodium salt (WST-8) assay as previously described.^81^

### Cellular reactive oxygen species (ROS) assay

ROS were detected using 2′,7′-dichlorofluorescin diacetate (DCFDA) cellular ROS detection assay kit (Abcam # ab113851) following the manufacturers' protocol. Cells were plated overnight in 96-well plates in their medium with or without metal complex. The fluorescence excitation/emission 485/535 was measured using the Synergy HTX Multi-Mode Microplate Reader (Bio-Tek).

### Statistical analysis

Statistical analysis was performed using two-tailed paired *t* test, one way ANOVA-Dunnett's test, and nonlinear regression analysis in GraphPad Prism 5.01 Software (San Diego, CA, USA).

### Western blot analysis

Cells were seeded in a 6-well plate and cultured in a 37 °C, 5.0% CO_2_ incubator to adhere to the cells. The old culture medium was discarded and replaced with a fresh medium containing 50 μM of the iron complex. Incubation was then continued for 24 h. After treatment was completed, the cells were washed with PBS. The total protein was extracted using an RNA/protein extraction kit (MACHEREY-NAGEL) according to the manufacturer's instructions. Lysates (50 μg) were subjected to 8–12% sodium dodecyl sulfate-polyacrylamide gel (SDS-PAGE), and then proteins were transferred to polyvinylidene fluoride (PVDF) membranes. Primary antibodies diluted 1% skim milk in PBS with 1.0% Tween-20 (Sigma) and then incubated at 4 °C overnight. Primary antibody: caspase-9 (cell signaling technology (CST), 9508), cleaved caspase-9 (CST, 7237), caspase-3 (CST, 9662), cleaved caspase-3 (CST, 9661), PARP (CST, 9542), cleaved PARP1 (CST, 5625), β-actin (bethyl laboratories, A300-491A). The horseradish peroxidase-conjugated secondary antibodies in 2.0% skim milk in PBS with 1.0% Tween-20 were incubated at room temperature for 1 h. Signals were induced using the chemiluminescent detection reagent (ATTO) and detected using an Amersham Imager 600 (GE Healthcare).

### Immunocytochemistry

Cells were fixed with 4% paraformaldehyde and permeabilized with 0.20% Triton X-100 in phosphate-buffered saline (PBS). Following fixation, cells were incubated at 4 °C overnight with anti-COX IV and anti-E-cadherin primary antibody in PBS with 1.0% bovine serum albumin (BSA) and 0.20% Triton X-100. The stained proteins were visualized using Alexa Fluor 488 and Alexa Fluor 594-conjugated secondary antibodies. The nuclei were counterstained with DAPI. The stained cells were observed with a LSM 700 Confocal Laser Scanning Microscope (Zeiss). The nuclei area and number of nuclei were measured by the ImageJ software (NIH).

### Quantitative reverse transcription-polymerase chain reaction (qRT-PCR)

The total RNA was extracted using an RNA/Protein extraction kit (MACHEREY-NAGEL). cDNA was synthesized from total RNA (2 μg) using M-MLV Reverse Transcriptase (Promega, M1705). qRT-PCR was performed on a LightCycler 96 System (Roche) using qPCRBIO SyGreen Blue Mix (PCR Biosystems, PB20.15), according to the manufacturer's instructions. The following targets were amplified using the indicated primer pairs (Table S3†).

### *In vivo* experiments

All animal experiments were performed according to the guidelines of the Chonnam National University Medical School Research Institutional Animal Care Committee, and all experimental protocols were approved by the committee. Five–six-week-old Balb/c-nu mice were obtained from OrientBio, Inc. (Seongnam, Korea) and housed in metal cages with free access to water and food. To generate the subcutaneous colon cancer models, HT29 human colon cancer cells were harvested during exponential growth and resuspended in a 1 : 1 mixture of saline and Matrigel (BD Biosciences). Next, 1 × 10^7^ HT29 cells per mouse were injected subcutaneously into the flank of Balb/c-nu mice. Mice were randomly assigned to three groups (*N* = 3): sterile distilled water (control), 1 (concentration: 1 mg kg^−1^), and 1 (concentration: 5 mg kg^−1^). When the average tumor size reached a volume of approximately 100 mm^3^, 1 was administered *via* intraperitoneal injections five times for 5 days. Tumor growth was monitored every 2 to 3 days; mice were monitored for signs of toxicity, and the size of the tumors were measured.

## Author contributions

YL, CO and JK designed and conducted all the experiments, and analyzed the data. M.-S. P. and W. K. B. performed *in vivo* study. SH and KHY drafted the manuscript. SH and KHY provided overall guidance throughout the process including experimental design, data analysis, and manuscript preparation. SH and KHY also revised the manuscript.

## Conflicts of interest

There are no conflicts to declare.

## Supplementary Material

SC-013-D1SC05094J-s001

SC-013-D1SC05094J-s002
