# Supplementary material for: Bioinspired nonheme iron complex that triggers mitochondrial apoptotic signalling pathway specifically for colorectal cancer cells
Source: Chem Sci. 2021 Dec 11;13(3):737–47. doi: 10.1039/d1sc05094j (PMC8768841; doi:10.1039/d1sc05094j)
Supplement: SC-013-D1SC05094J-s001 [file SC-013-D1SC05094J-s001.pdf]

Electronic Supplementary Material (ESI) for Chemical Science.
This journal is © The Royal Society of Chemistry 2021

Electronic Supplementary Information

Bioinspired nonheme iron complex that triggers mitochondrial apoptotic signalling pathway specifically for colorectal cancer cells

Yool Lee,[#a] Chaeun Oh,[#b] Jin Kim,[c] Myong-Suk Park,[d] Woo Kyun Bae,*[de] Kyung Hyun Yoo*[b] and Seungwoo Hong*[a]

[a] Department of Chemistry, Sookmyung Women's University, Seoul 04310, Korea.

[b] Department of Biological Sciences, Sookmyung Women's University, Seoul 04310, Korea.

[c] Department of Chemistry, Sunchon National University, Suncheon 57922, Korea.

[d] Division of Hemato-Oncology, Department of Internal Medicine, Chonnam National University Medical School and Hwasun Hospital, Hwasun, Republic of Korea.

[e] Combinatorial Tumor Immunotherapy MRC Center, Chonnam National University Medical School, Hwasun, Republic of Korea.

[#] *These authors contributed equally to this work.*

* To whom correspondence should be addressed.

E-mail: drwookyun@jnu.ac.kr; khryu@sookmyung.ac.kr, hsw@sookmyung.ac.kr

Table of Contents

Fig. S1	S3
Fig. S2	S4
Fig. S3	S5
Fig. S4	S6
Fig. S5	S7
Fig. S6	S8
Fig. S7	S9
Fig. S8	S10
Fig. S9	S11
Fig. S10	S12
Fig. S11	S13
Fig. S12	S14
Fig. S13	S15
Fig. S14	S16
Table S1	S17
Table S2	S18
Table S3	S19

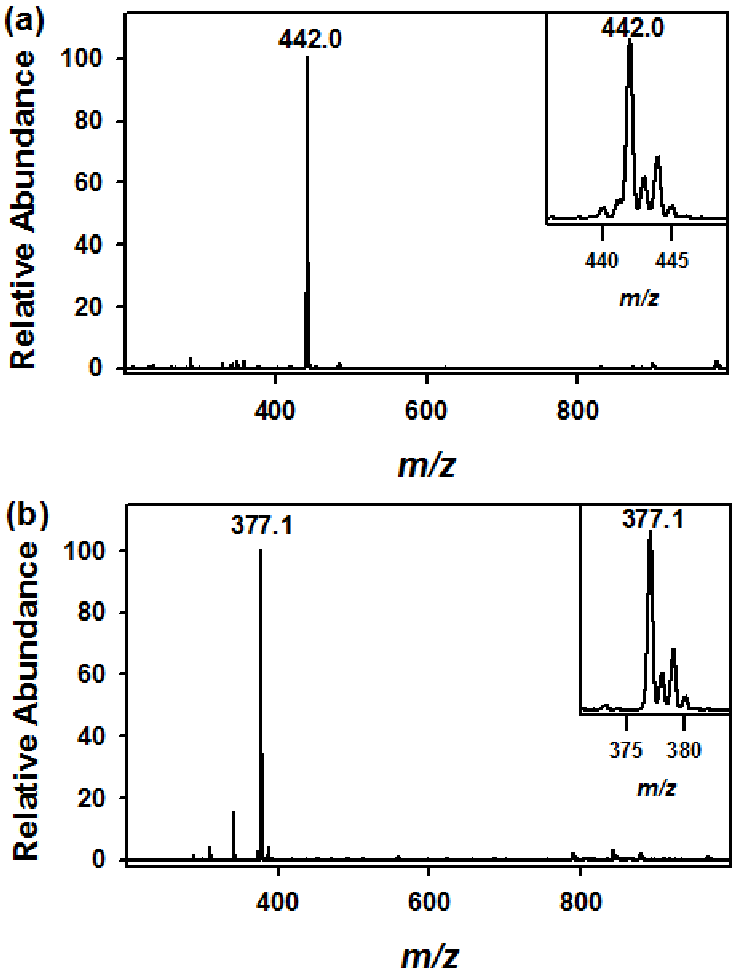

Fig. S1. ESI MS spectra of (a) **1**, and (b) [Mn(HN3O2)$(Cl)_2$] recorded in CH_3CN. The prominent ion peak at *m/z* of (a) 442.0, and (b) 377.1 correspond to $[Fe(HN3O2)(ClO4)]^+$ (calculated *m/z* of 442.1) and $[Mn(HN3O2)(Cl)]^+$ (calculated *m/z* of 377.1), respectively. Insets show the observed isotopic distribution patterns of **1**, and [Mn(HN3O2)$(Cl)_2$].

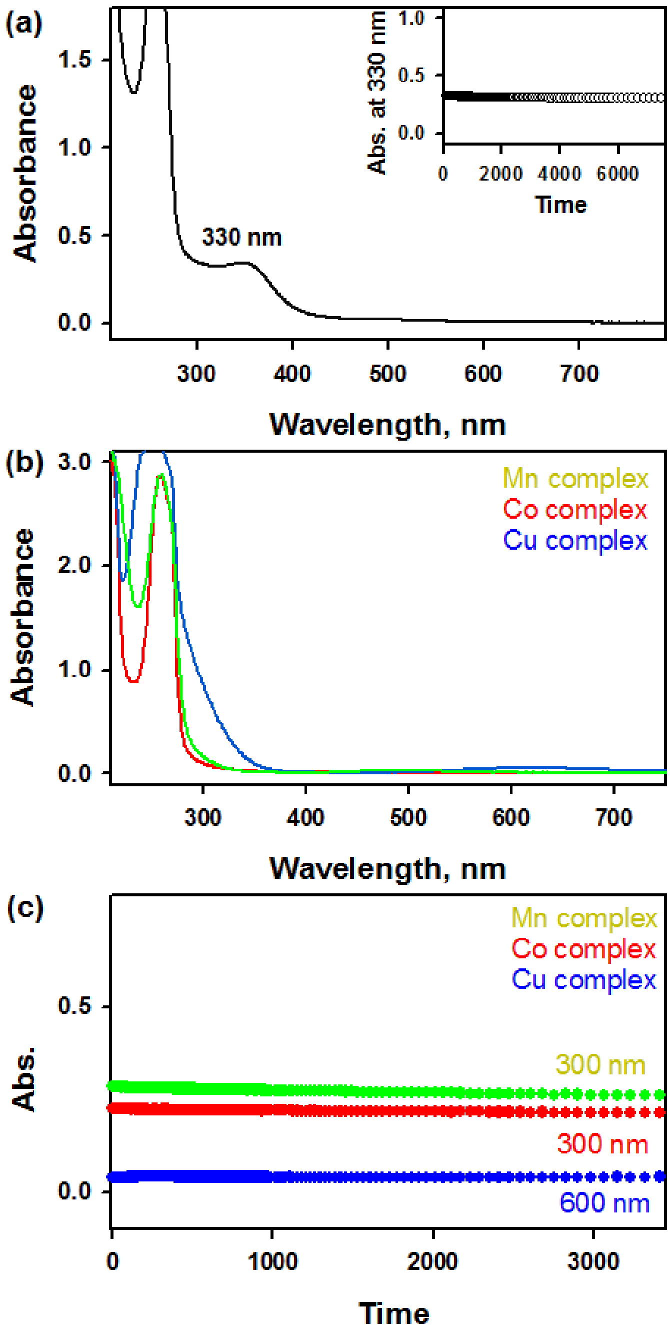

Fig. S2. (a) UV-vis spectrum of **1** (0.25 mM) in CH_3CN at 20 °C. Inset shows the time course monitored at 330 nm for the stability of **1**. (b) UV-vis spectra of $[Mn(HN3O2)(Cl)_2]$ (0.25 mM, green line), $[Co(HN3O2)]^{2+}$ (0.25 mM, red line) and $[Cu(HN3O2)]^{2+}$ (0.25 mM, blue line) in CH_3CN at 20 °C. (c) Time courses monitored at 300 nm for $[Mn(HN3O2)(Cl)_2]$ (green circle), $[Co(HN3O2)]^{2+}$ (red circle) and 600 nm for $[Cu(HN3O2)]^{2+}$ (blue circle).

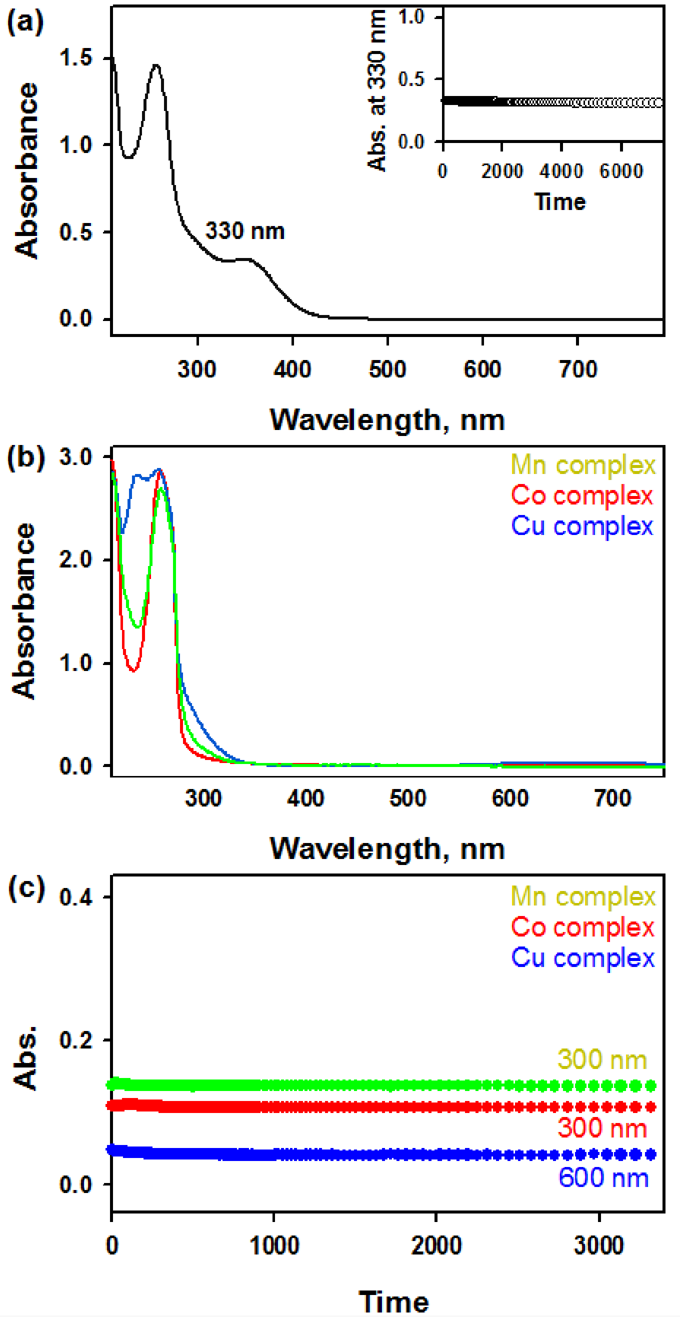

Fig. S3. (a) UV-vis spectrum of **1** (0.25 mM) in H_2O at 20 °C. Inset shows the time course monitored at 330 nm for the stability of **1**. (b) UV-vis spectra of [Mn(HN3O2)(Cl)$_2$] (0.25 mM, green line), $[Co(HN3O2)]^{2+}$ (0.25 mM, red line) and $[Cu(HN3O2)]^{2+}$ (0.25 mM, blue line) in H_2O at 20 °C. (c) Time courses monitored at 300 nm for [Mn(HN3O2)(Cl)$_2$] (green circle), $[Co(HN3O2)]^{2+}$ (red circle) and 600 nm for $[Cu(HN3O2)]^{2+}$ (blue circle).

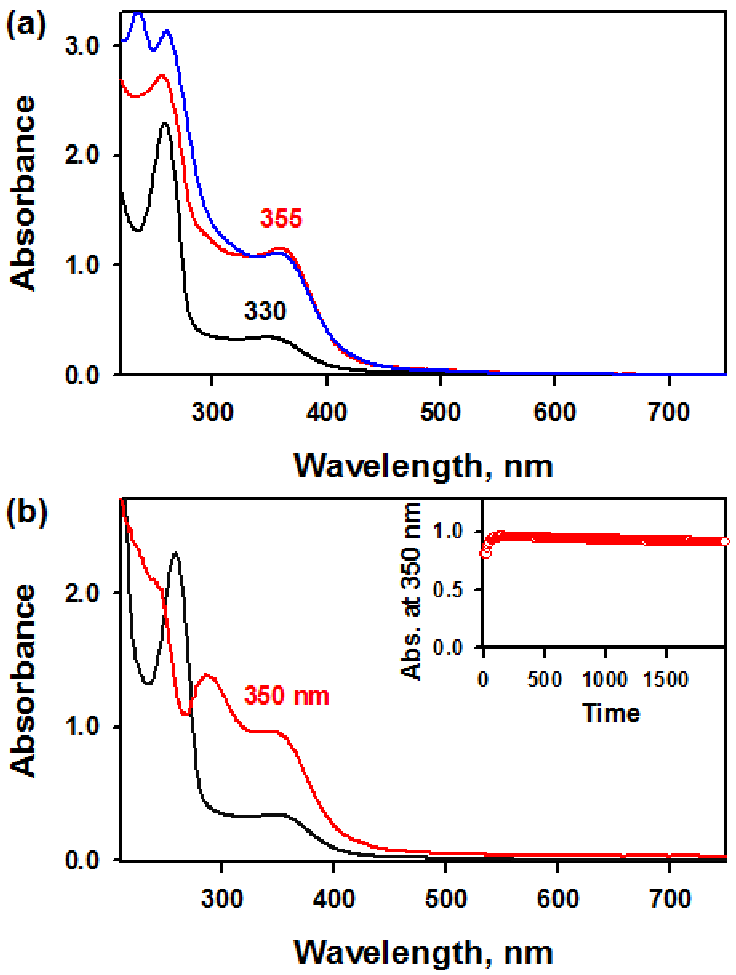

Fig. S4. (a) UV-vis spectra of **2** (0.20 mM) obtained in the reaction of **1** (0.20 mM) with PhIO (0.40 mM, blue line) and H_2O_2 (0.60 mM, red line) in CH_3CN at 20 °C. (b) UV-vis spectrum of **2** obtained in the reaction of **1** (0.20 mM) with H_2O_2 (0.60 mM, red line) in CH_3CN:H_2O (v/v 1:1) at 5 °C.

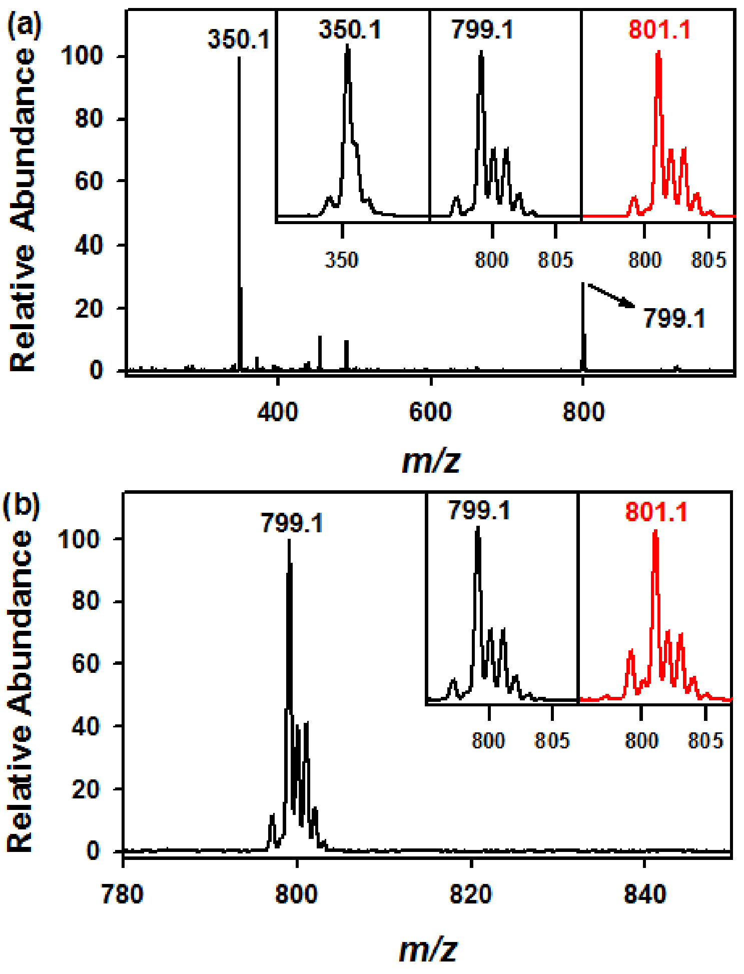

Fig. S5. ESI MS spectra of **2** obtained (a) in the reaction between **1** (0.20 mM) and PhI$^{16/18}$O (0.40 mM) in CH_3CN at 20 °C and (b) in the dioxygen activation reaction of **1** in the presence of BNAH, and $HClO_4$ in $^{16}O_2$ and $^{18}O_2$-saturated CH_3CN at 20 °C. The prominent ion peak at *m/z* of 350.1, and 799.1 correspond to $[Fe_2(O)(N3O2)_2]^{2+}$ (calculated *m/z* of 350.1) and $[Fe_2(O)(N3O2)_2(ClO_4)]^+$ (calculated *m/z* of 799.1), respectively. Insets show the observed isotopic distribution patterns of $[Fe_2(O)(N3O2)_2]^{2+}$, $[Fe_2(^{16}O)(N3O2)_2(ClO_4)]^+$ and $[Fe_2(^{18}O)(N3O2)_2(ClO_4)]^+$.

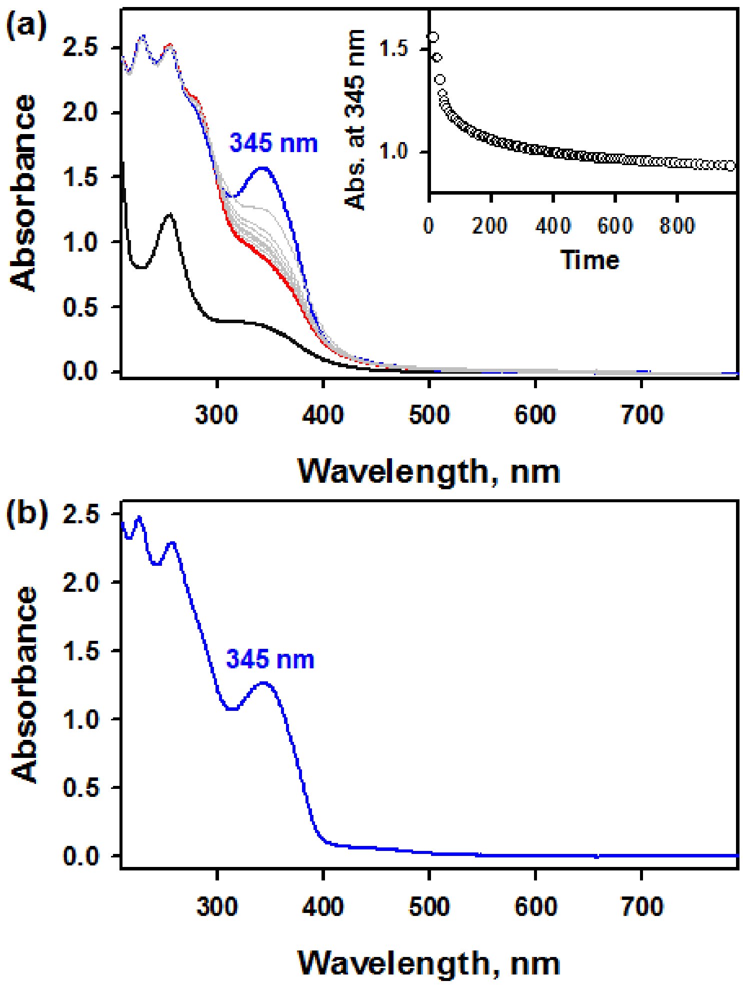

Fig. S6. (a) UV-Vis spectral changes observed in the reaction of **1** (black line, 0.10 mM) and BNAH (blue line, 0.20 mM) in the presence of $HClO_4$ (0.10 mM) in air-saturated CH_3CN at 20 °C. The use of hydrochloric acid instead of $HClO_4$ showed the identical spectrum (data not shown). Inset shows the time courses monitored at 345 nm due to the decay of BNAH. (b) UV-vis spectrum of BNAH (0.20 mM) in air-saturated CH_3CN at 20 °C.

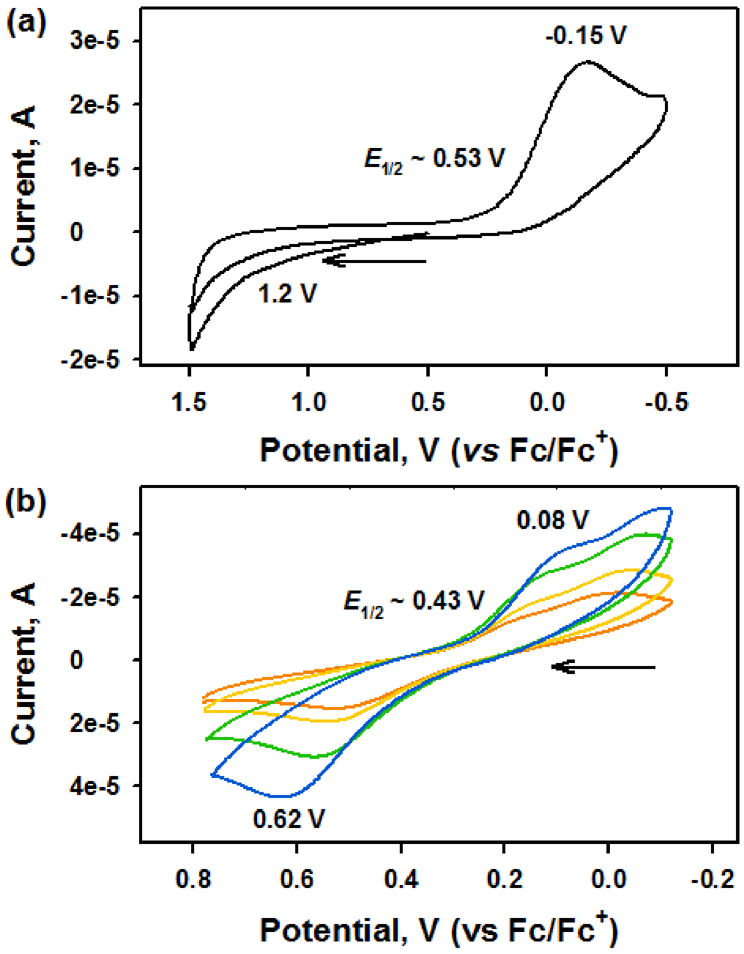

Fig. S7. Cyclic voltammogram of (a) **1** (2.0 mM) in CH_3CN containing $TBAPF_6$ (0.10 M) with a glassy carbon working electrode at 20 °C with a scan rate of 0.10 V s^{-1} and (b) **1** (4.0 mM) in H_2O containing $NaClO_4$ (0.10 M) with a glassy carbon working electrode at 20 °C with scan rates of 0.10 (orange line), 0.20 (yellow line), 0.50 (red line), and 1.0 (blue line) V s^{-1}.

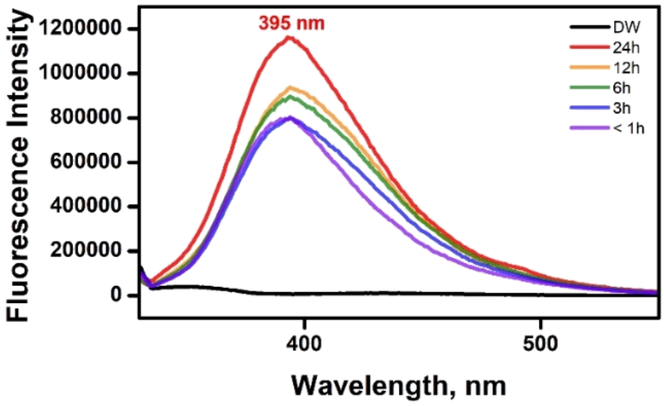

Fig. S8. Fluorescence intensity changes obtained in the reaction between **1** and TA in DW at room temperature.

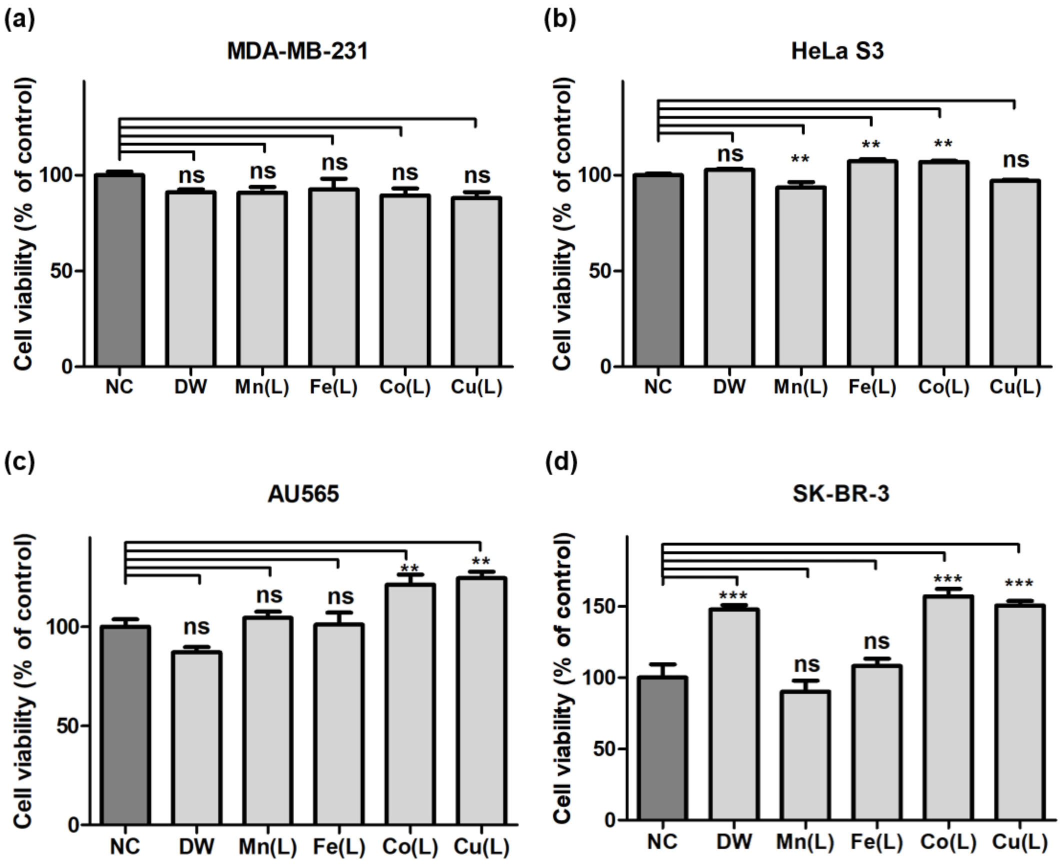

Fig. S9. Effects of treatment of negative control (NC), deionized water (DW), and $[M(HN3O2)]^{2+}$ (M(L); M = Mn, Fe, Co, and Cu) on (a) MDA-MB-231 (b) HeLa S3 (c) AU565 (d) SK-BR-3 cells by WST-8 assay with respect to total cell viability after 24h. The viability of cells without additional complexes is defined as 100%. The statistical analysis was performed using one way ANOVA-Dunnett's test (* $p < 0.05$, ** $p < 0.01$, *** $p < 0.001$ and ns = nonsignificant as compared to control).

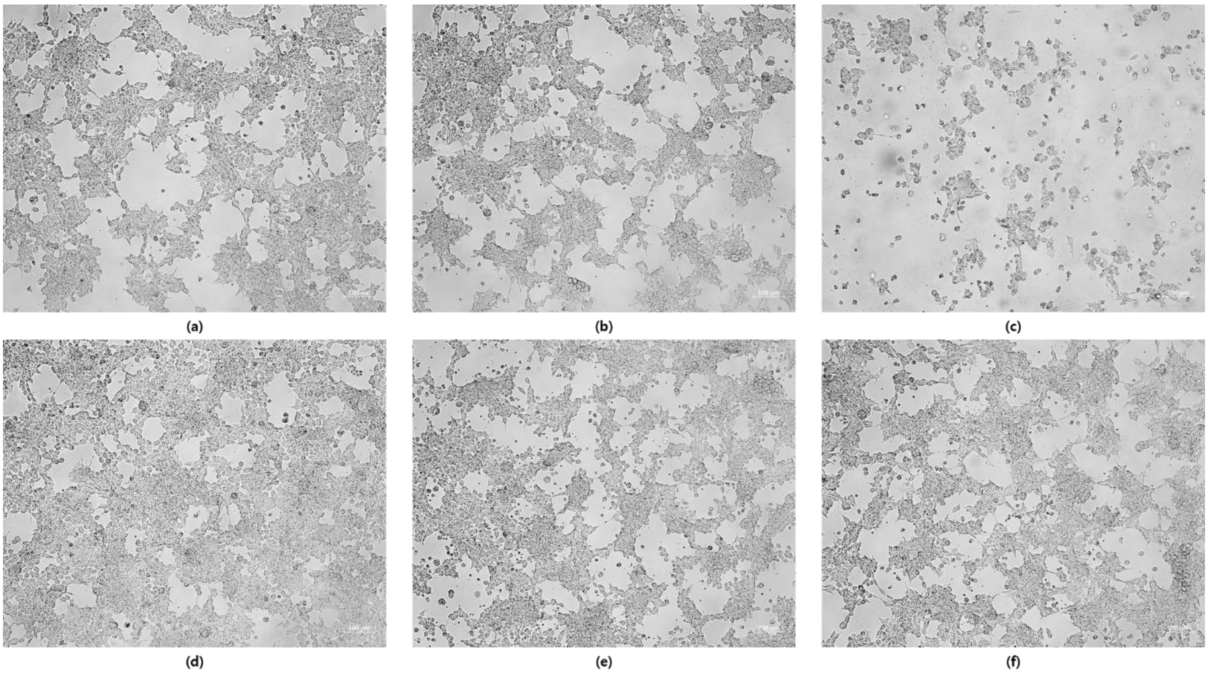

Fig. S10. Representative images of HCT116 cells (a) negative control, (b) deionized water, and after incubation with (c) **1**, (d) $[Mn(HN3O2)]^{2+}$, (e) $[Co(HN3O2)]^{2+}$, and (f) $[Cu(HN3O2)]^{2+}$ for 24 h. Scale bar: 100 μm.

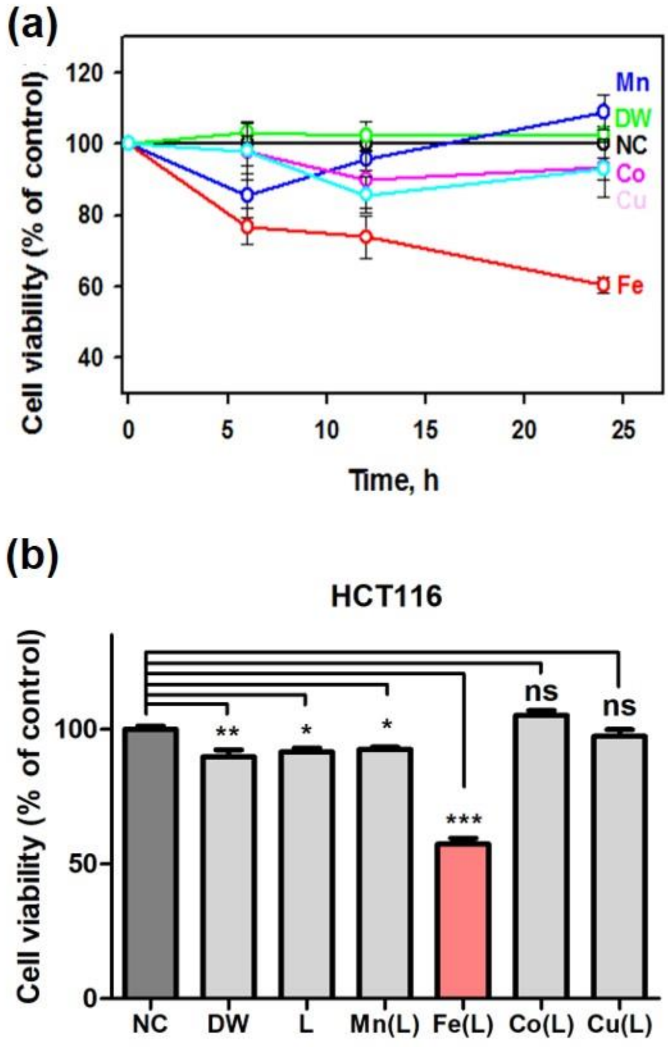

Fig. S11. Effects of treatment of deionized water (DW, green), $[M(HN3O2)]^{2+}$ (M(L); M = Mn (blue), Fe (red), Co (pink), Cu (cyan)), and ligand on HCT116 cells by WST-8 assay (a) with respect to time and (b) total cell viability after 24 h. The viability of HCT116 cells without additional complexes is defined as 100%. The statistical analysis was performed using one way ANOVA-Dunnett's test (*** = $p < 0.0001$; ns = nonsignificant as compared to control).

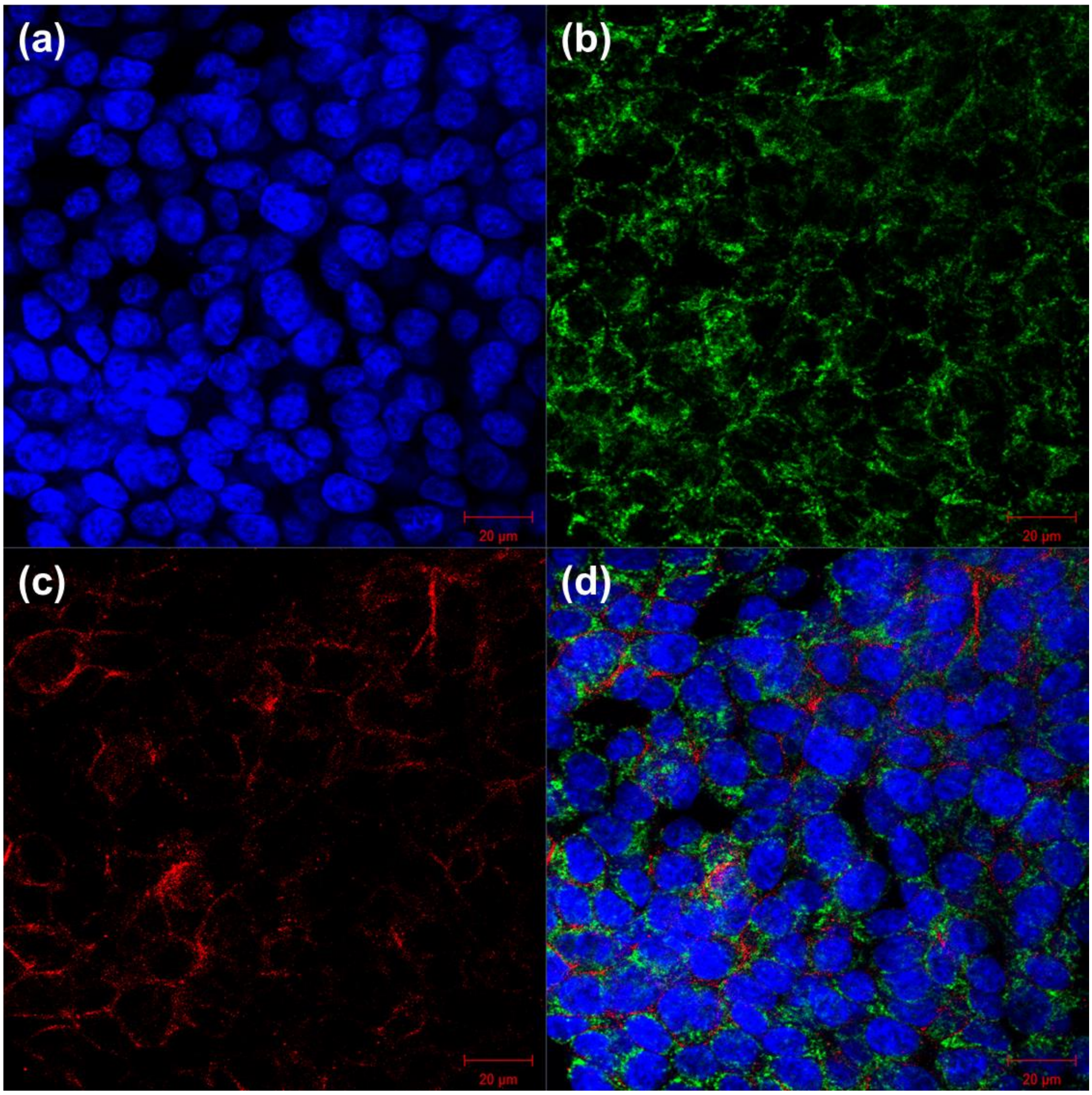

Fig. S12. Controlled confocal fluorescence images of HCT116 cells after 24 h incubation with DW followed by staining with (a) DAPI (blue), (b) antibody-COX IV (green), (c) antibody-E-cadherin (red), and (d) merged image. Scale bar: 20 μm.

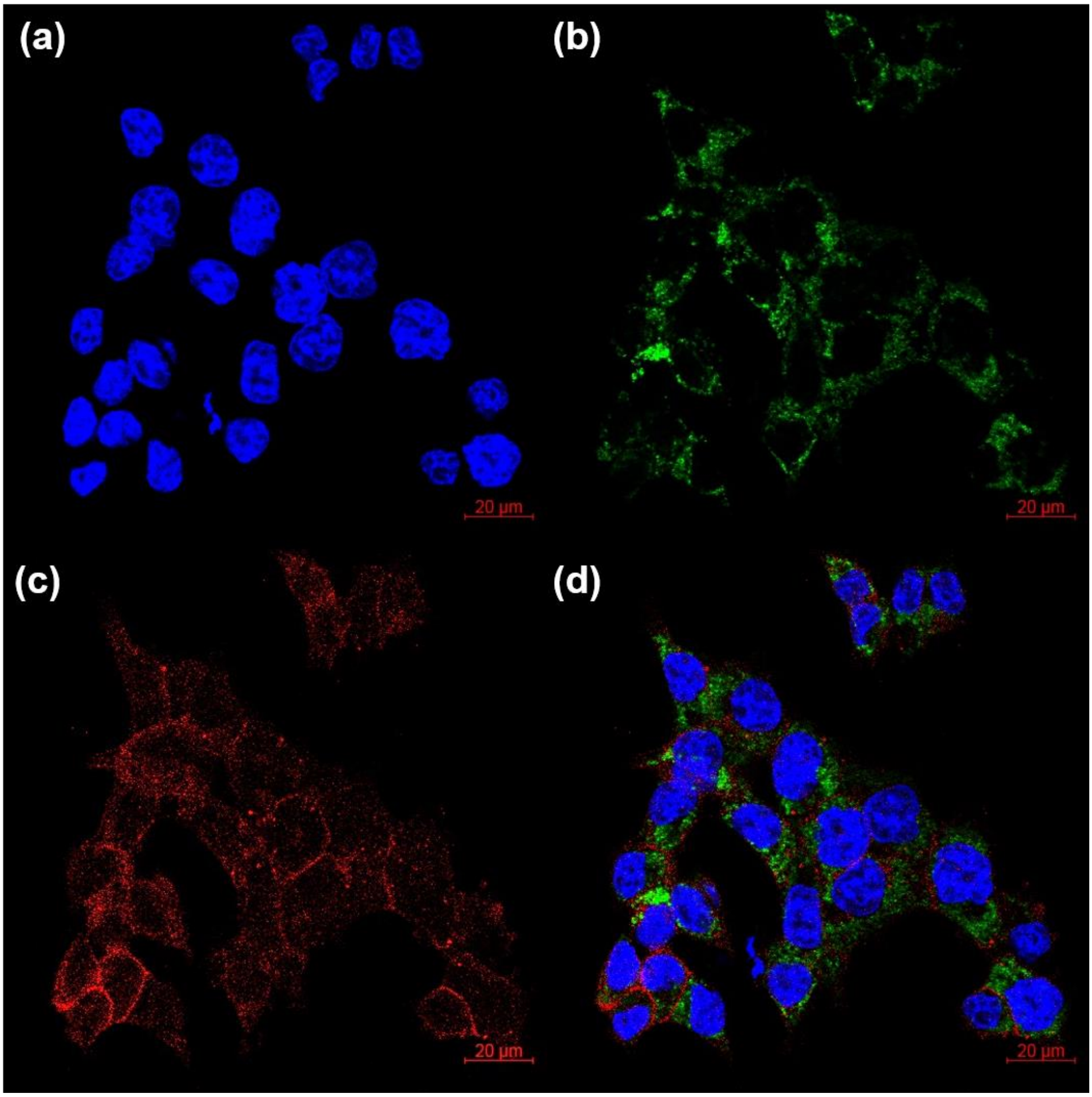

Fig. S13. Confocal fluorescence images of HCT116 cells after 24 h incubation with **1** followed by staining with (a) DAPI (blue), (b) antibody-COX IV (green), (c) antibody-E-cadherin (red), and (d) merged image. Scale bar: 20 μm.

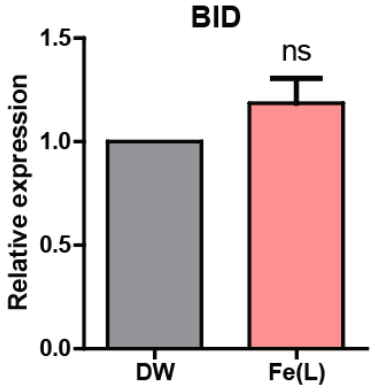

Fig. S14. The expression level of BID in HCT116 cells treated with **1**.

Table S1. Crystallographic data and refinements for [Mn(HN3O2)(Cl)$_2$], **1**, and **2**.

	[Mn(HN3O2)(Cl)$_2$]	**1**	**2**
Empirical formula	$C_{16}H_{21}Cl_2MnN_3O_2$	$C_{16}H_{25}Cl_2FeN_3O_{12}$	$C_{32}H_{46}Cl_4Fe_2N_6O_{23}$
Formula weight	413.20	578.41	1136.25
Temperature (K)	120	120	120
Wavelength (Å)	0.71073	0.71073	0.71073
Crystal system/space group	orthorhombic, P_{na21}	monoclinic, P_{21}	monoclinic, $P_{c2/c}$
Unit cell dimensions			
a (Å)	14.289(4)	14.194(4)	15.9079(12)
b (Å)	8.933(2)	10.712(3)	17.3412(13)
c (Å)	28.022(7)	15.769(4)	16.1343(12)
α (°)	90	90	90
bβ (°)	90	102.510(4)	90.724(1)
γ (°)	90	90	90
Volume (Å ^{3})	3576.8(16)	2340.8(11)	4450.5(6)
Z	8	4	4
Calculated density (g/cm^{-3})	1.535	1.641	1.696
Absorption coefficient (mm^{-1})	1.051	0.939	0.985
Reflections collected	6188	7959	3913
Absorption correction	multi-scan (T_{min} = 0.591, T_{max}= 0.745)	multi-scan (T_{min} = 0.601, T_{max}= 0.745)	multi-scan (T_{min} = 0.689, T_{max}= 0.745)
Independent reflections	5717	6552	3589
Goodness-of-fit on F^2	1.097	1.017	1.082
R [F^2 > 2sigma(F^2)]	0.0404	0.0359	0.0298
wR^2	0.1052	0.0864	0.0817

Table S2. Selected bond distances (Å) and angles (°) for [Mn(HN3O2)(Cl)$_2$], **1**, and **2**.

	[Mn(HN3O2)(Cl)$_2$]	**1**	**2**
Bond Distances (Å)			
M1-N1	2.255(4)	2.118(4)	2.2167(18)
M1-N2	2.341(4)	2.252(4)	2.1813(17)
M1-N3	2.248(4)	2.122(3)	2.1431 (17)
M1-O1	2.358(4)	2.158(3)	2.1813(17)
M1-O2	-	2.068(3)	2.0016(15)
M1-O3$_{\text{(H2O or bridging)}}$	-	2.148(3)	1.7799(4)
M1-M2	2.4054(13)	-	3.530(1)
M1-Cl1	2.4054(13)	-	-
M1-Cl2	2.4580(14)	-	-
Bond Angles (°)			
N1-M1-N2	72.73(13)	77.59(14)	77.39(6)
N1-M1-N3	146.48(14)	150.88(14)	154.99(7)
N1- M1-O1	82.61(13)	95.35(14)	88.05(6)
N2-M1-O2	-	155.06(14)	153.37(6)
N1-M1-O3	-	85.04(14)	96.94(6)
O1-M1-O3	-	166.26(12)	174.52(6)
M1-O3-M2	-	-	165.08(6)
N2-M1-Cl1	163.21(11)	-	-
O1-M1-Cl2	165.23(9)	-	-
N1-M1-Cl1	108.05(11)	-	-
N1-M1-Cl2	92.31(11)	-	-

Table S3. Sequences of qRT-PCR primers.

Gene		Sequences (5’ → 3’)
hBCL2 alpha	Forward	ATGTGTGTGGAGAGCGTCAA
	Reverse	CCGTACAGTTCCACAAAGGC
hBCL2 beta	Forward	ATGTGTGTGGAGAGCGTCAA
	Reverse	GCCCAGACTCACATCACCAA
hBAX	Forward	TGGGCTGGACATTGGACTTC
	Reverse	AAAGTAGGAGAGGAGGCCGT
hBAK	Forward	AAAGTAGGAGAGGAGGCCGT
	Reverse	ATGGGACCATTGCCCAAGTT
hBID	Forward	AGCACAGTGCGGATTCTGT
	Reverse	CTCATCCCTGAGGCTGGAAC
h18s rRNA	Forward	GTCGGCGTCCCCCAACTTCT
	Reverse	CGTGCAGCCCCGGACATCTA
